# Supplementary material for: Single-cell analysis reveals new evolutionary complexity in uveal melanoma
Source: Nat Commun. 2020 Jan 24;11:496. doi: 10.1038/s41467-019-14256-1 (PMC6981133; doi:10.1038/s41467-019-14256-1)
Supplement: Supplementary file 1 — Supplementary Information [file 41467_2019_14256_MOESM1_ESM.pdf]

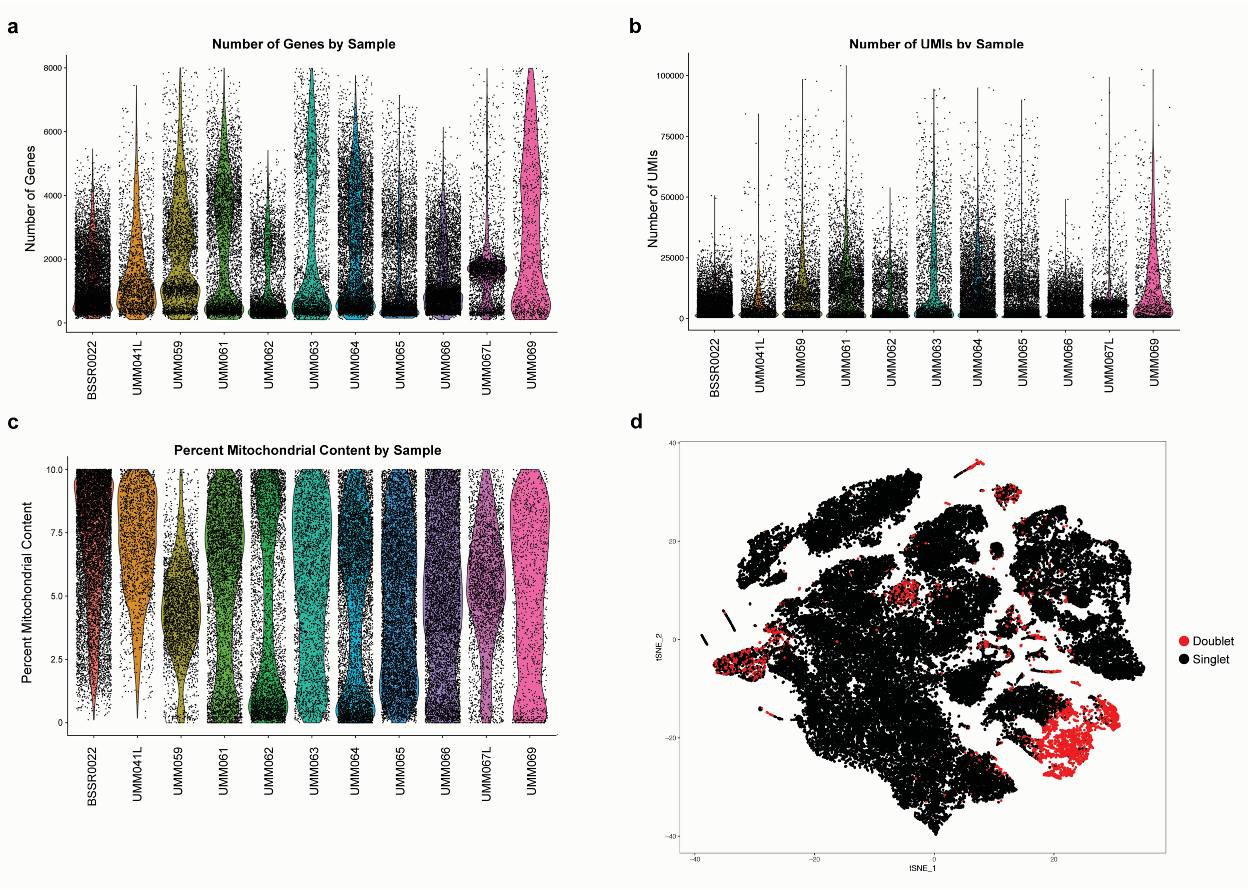

Supplementary Figure 1. Violin plots for scRNA-seq data quality control measures by Sample. a, Violin plot of Number of Genes by sample. **b,** Violin plot of Number of UMIs by sample. **c,** Violin plot of Percent Mitochondrial Content by sample. **d,** Combined t-distributed stochastic neighborhood embedding (tSNE) plot annotated by output of DoubletFinder analysis.

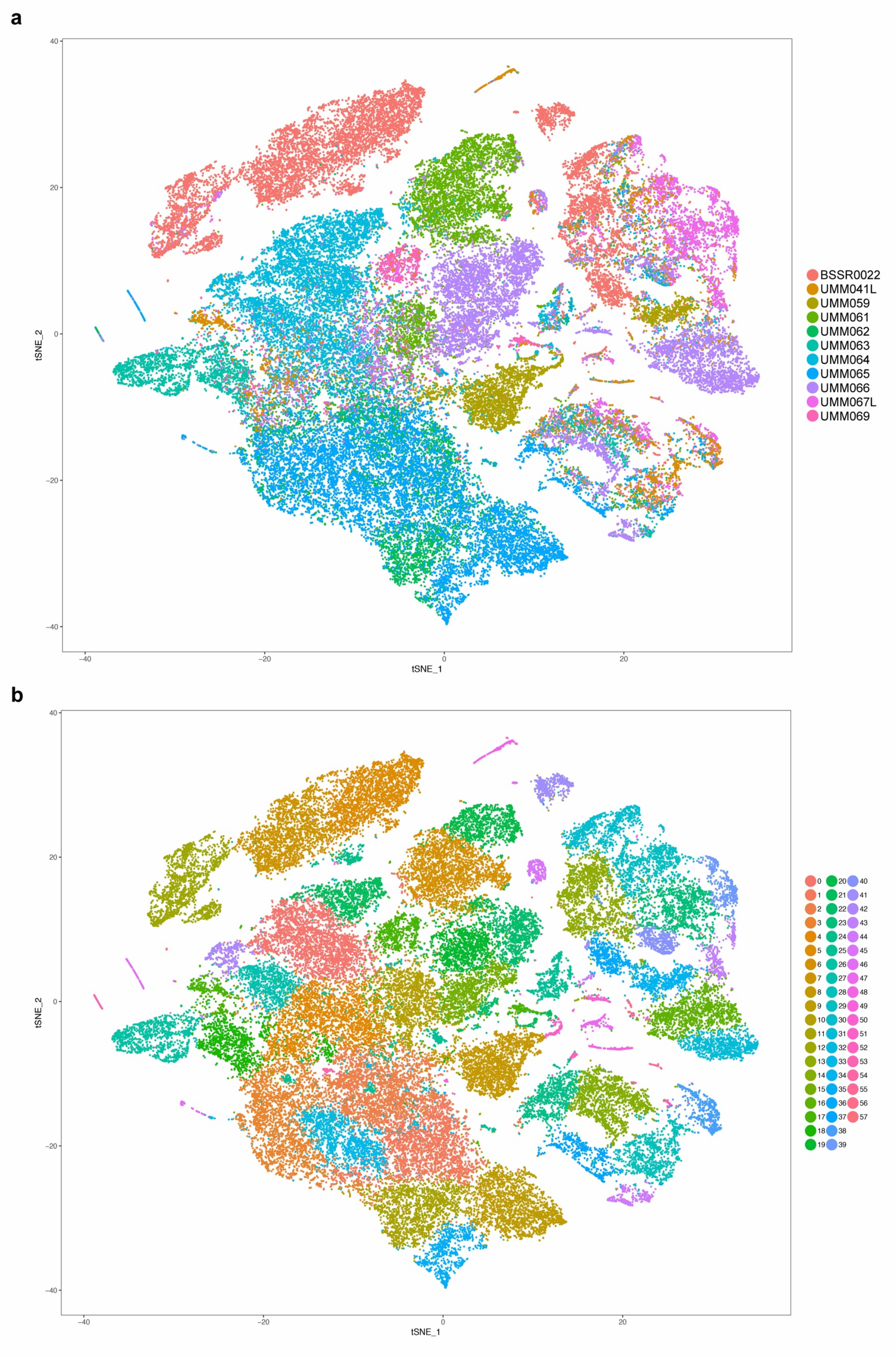

Supplementary Figure 2. Combined scRNA-seq analysis of all samples. a, Combined tSNE plot annotated by sample of origin. **b,** Combined tSNE plot annotated by Louvain cluster.

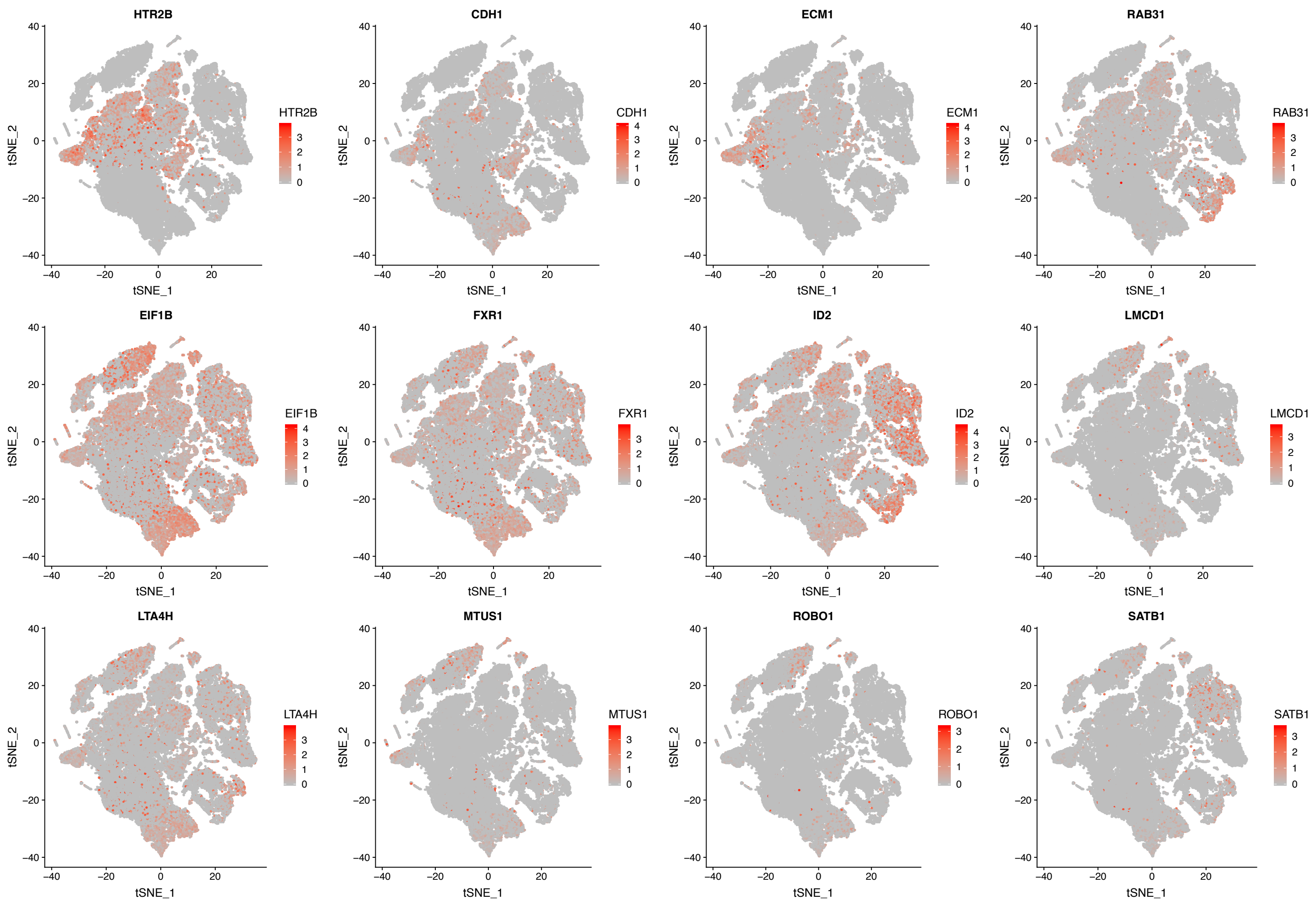

Supplementary Figure 3. Gene expression profile gene distribution in 8 primary and 3 metastatic uveal melanoma tumours. Combined t-distributed stochastic neighborhood embedding (tSNE) plots of the gene expression profile (GEP) genes showing expression in various tumour and immune cell types. Expression values are plotted as normalized counts.

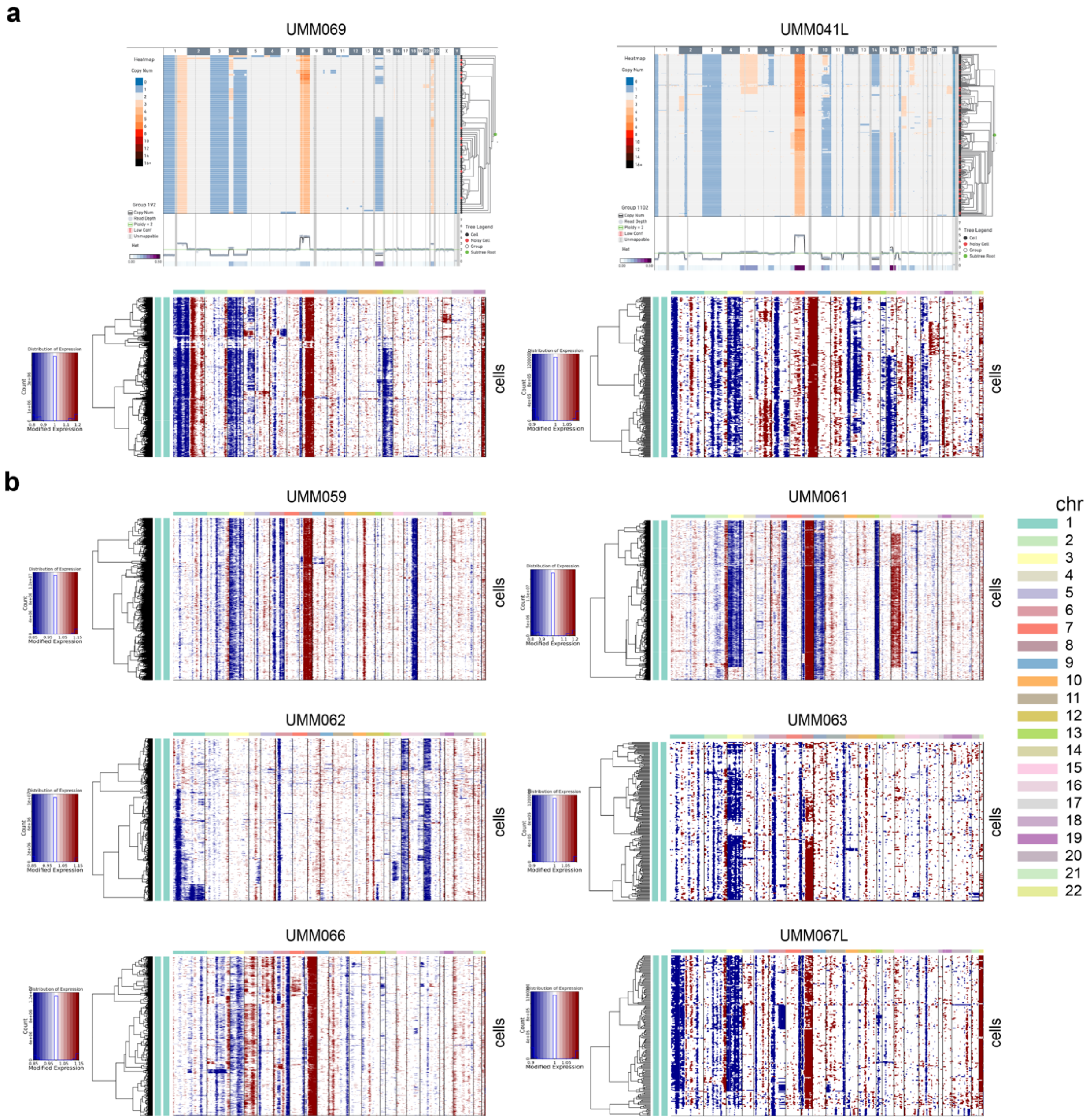

Supplementary Figure 4. Comparison of inferCNV analysis and scCNV analysis. a, Comparison of CNV profiles inferred from 5’ single cell gene expression data with single cell CNV data from patients UMM069 and UMM041L. **b,** inferCNV plots from patients UMM059, UMM061, UMM062, UMM063, UMM066, and UMM067L.

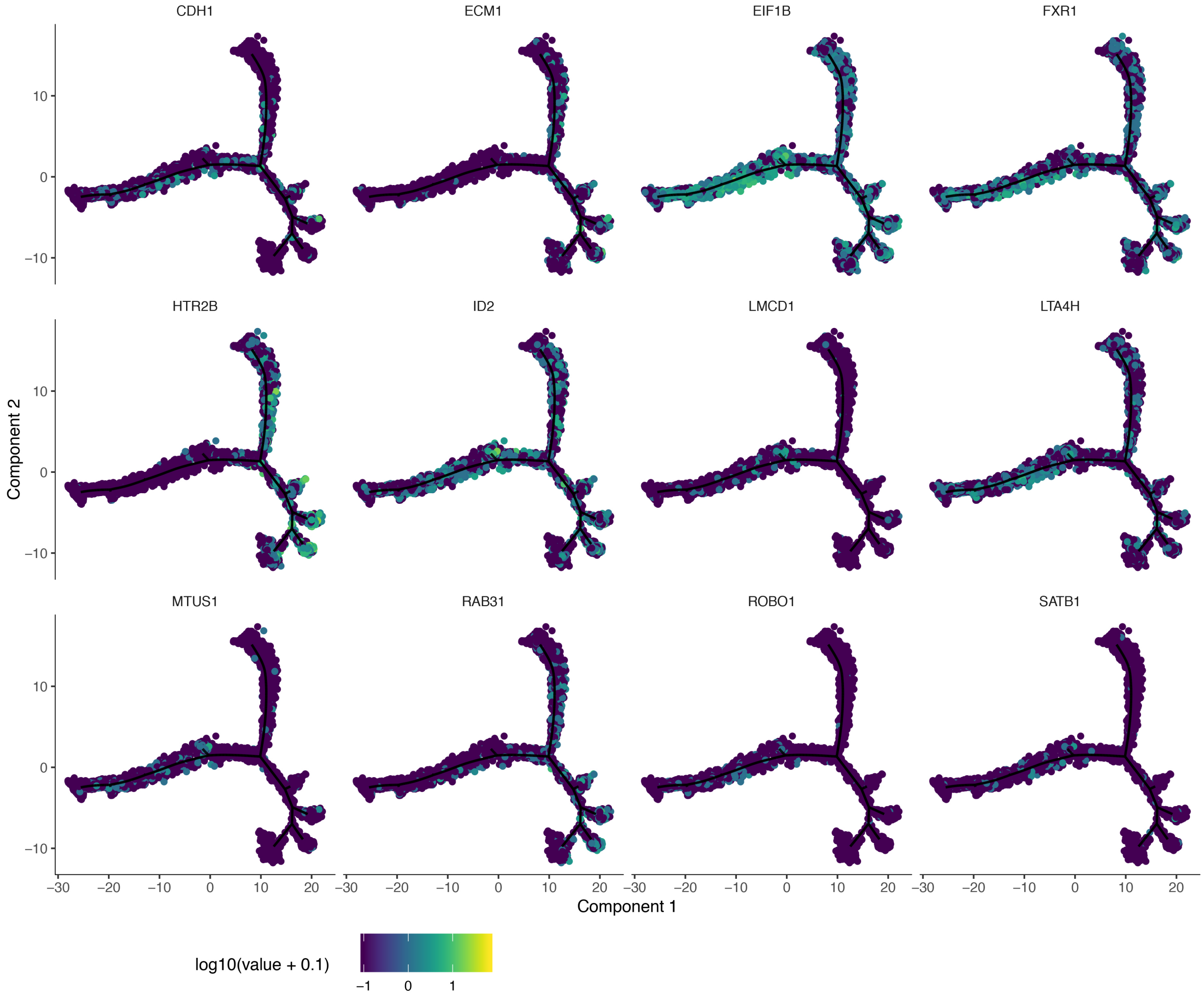

Supplementary Figure 5. Trajectory analysis of uveal melanoma cells with gene expression profile gene distribution. Monocle 2 trajectory analysis of 7,947 uveal melanoma cells obtained by 5' gene expression chemistry annotated by gene expression profile genes.

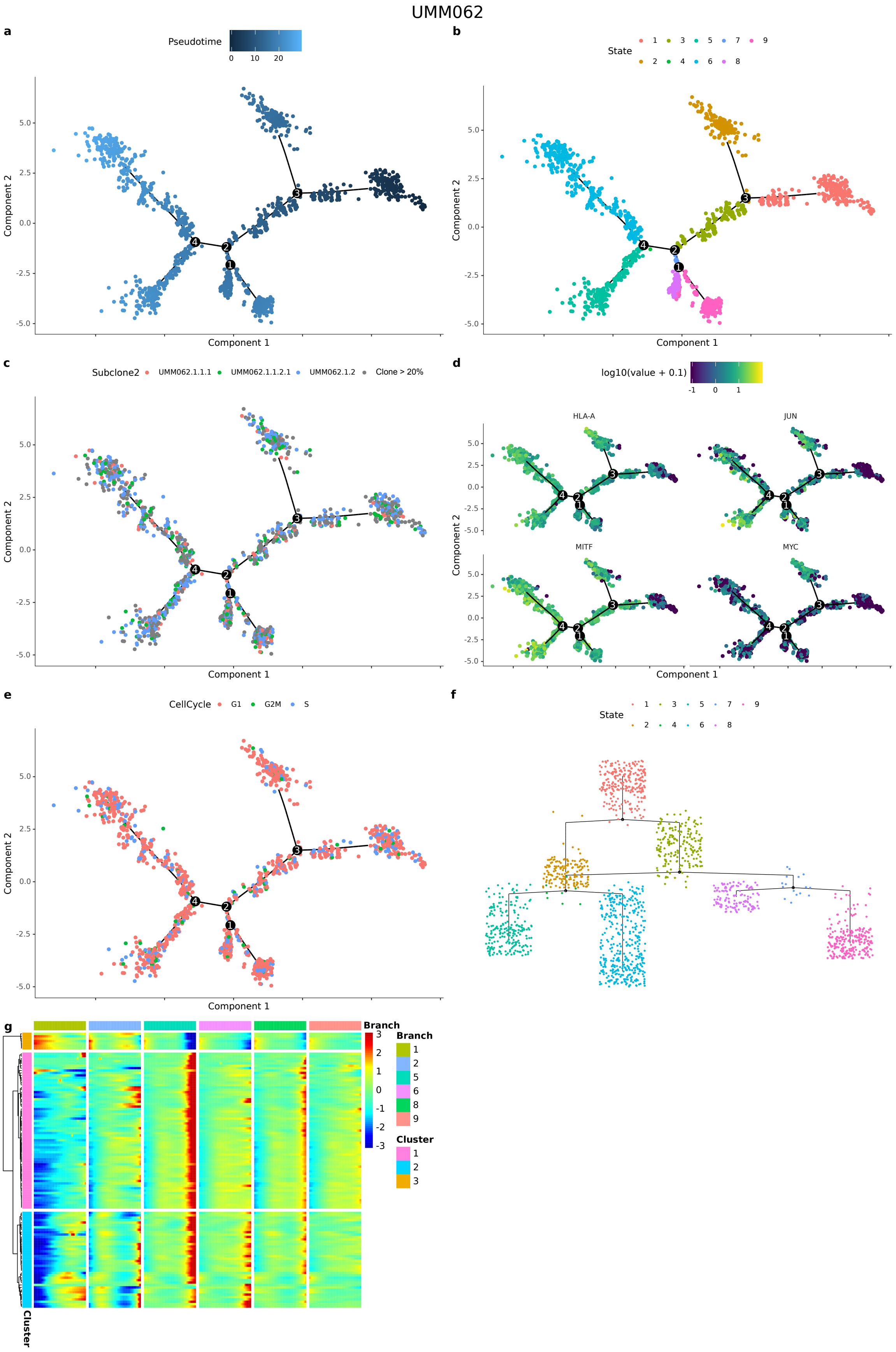

UMM062
a
Pseudotime
0 10 20
Component 2
Component 1
5.0
2.5
0.0
-2.5
-5.0
b
State
1 3 5 7 9
2 4 6 8
c
Subclone2
UMM062.1.1.1
UMM062.1.1.2.1
UMM062.1.2
Clone > 20%
d
log10(value + 0.1)
-1 0 1
HLA-A
JUN
MITF
MYC
e
CellCycle
G1
G2M
S
f
g
Branch
1
2
5
6
8
9
Cluster
1
2
3
3 2 1 0 -1 -2 -3

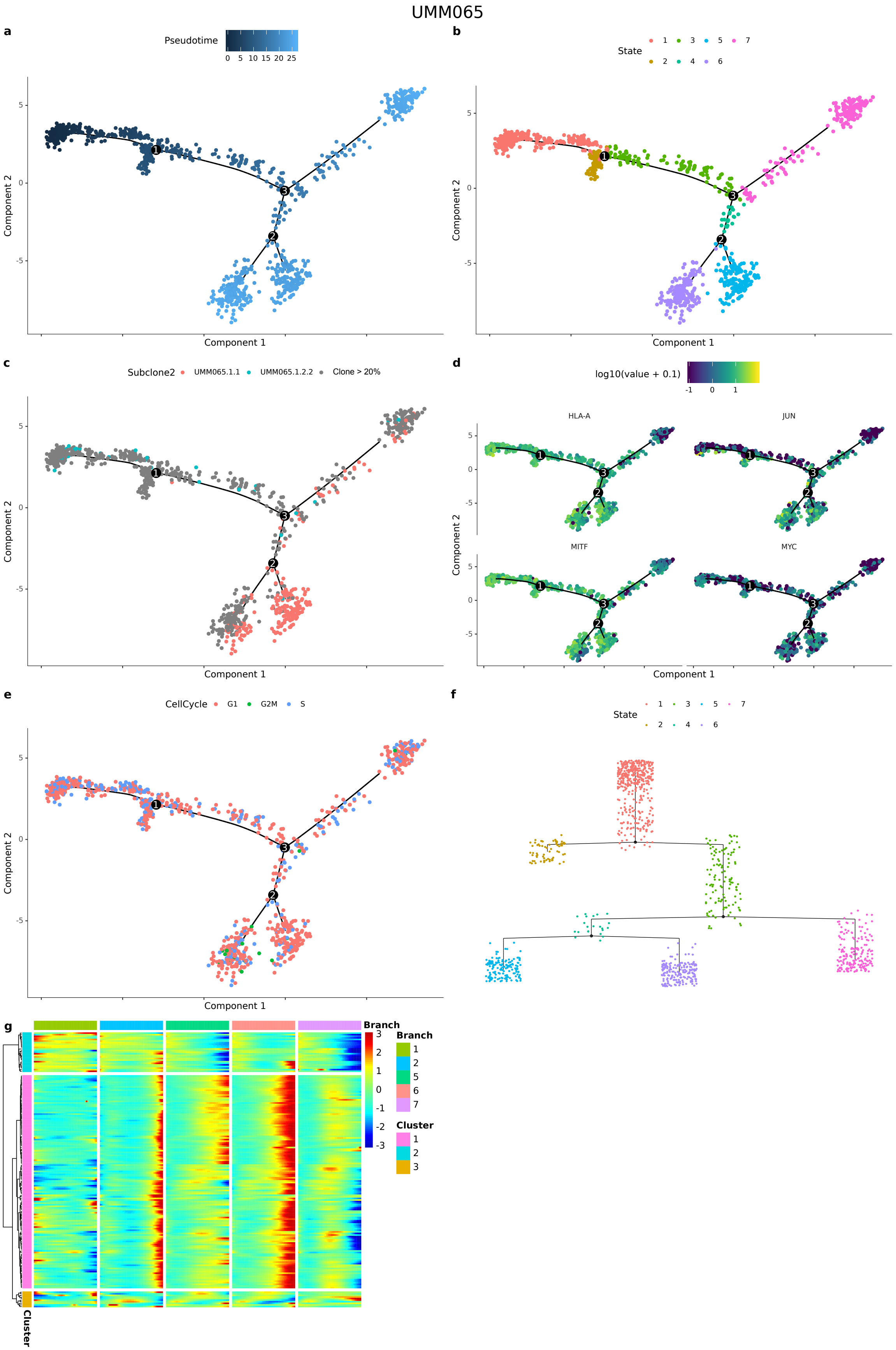
UMM065
a
Pseudotime
0 5 10 15 20 25
Component 1
Component 2
b
State
1 2 3 4 5 6 7
c
Subclone2
UMM065.1.1
UMM065.1.2.2
Clone > 20%
d
log10(value + 0.1)
HLA-A
JUN
MITF
MYC
e
CellCycle
G1
G2M
S
f
g
Branch
Cluster

BSSR0022

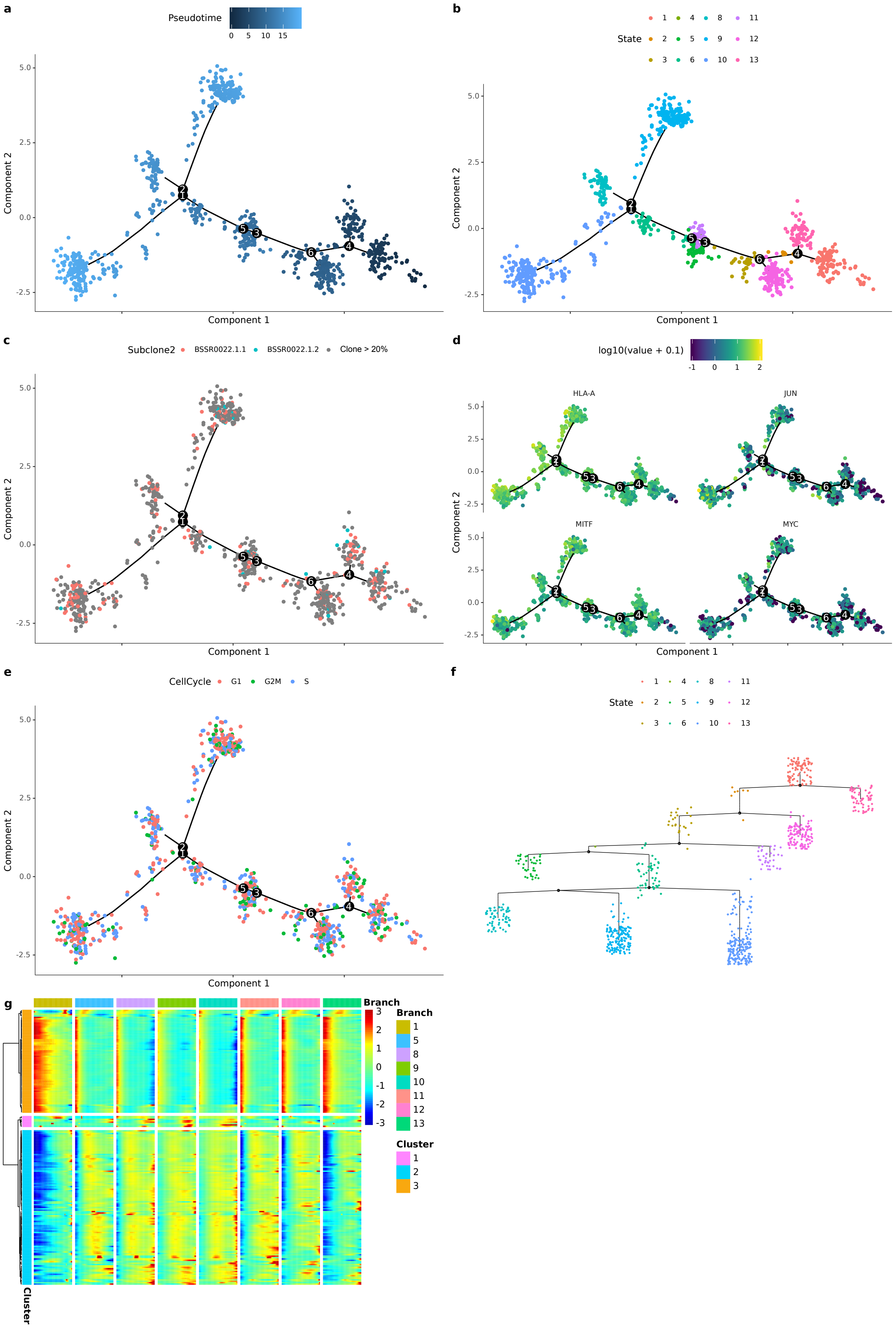
a
Pseudotime
0 5 10 15
Component 1
Component 2
b
State
1 2 3 4 5 6 8 9 10 11 12 13
c
Subclone2
BSSR0022.1.1
BSSR0022.1.2
Clone > 20%
d
log10(value + 0.1)
HLA-A
JUN
MITF
MYC
e
CellCycle
G1
G2M
S
f
g
Branch
Cluster

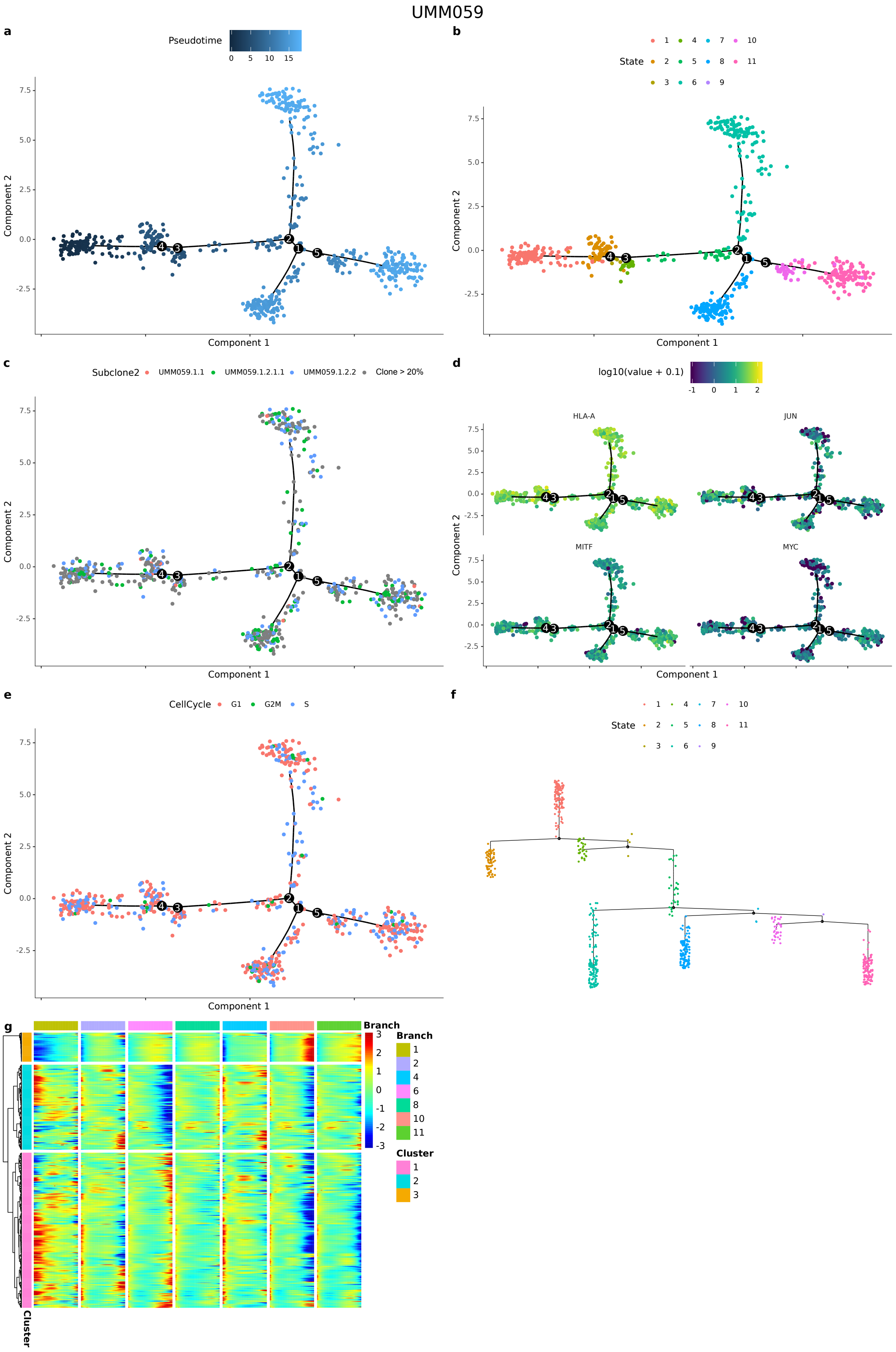
UMM059
a
Pseudotime
0 5 10 15
Component 1
Component 2
b
State
1 2 3 4 5 6 7 8 9 10 11
c
Subclone2
UMM059.1.1
UMM059.1.2.1.1
UMM059.1.2.2
Clone > 20%
d
log10(value + 0.1)
HLA-A
JUN
MITF
MYC
e
CellCycle
G1
G2M
S
f
g
Branch
1 2 4 6 8 10 11
Cluster
1 2 3

UMM061

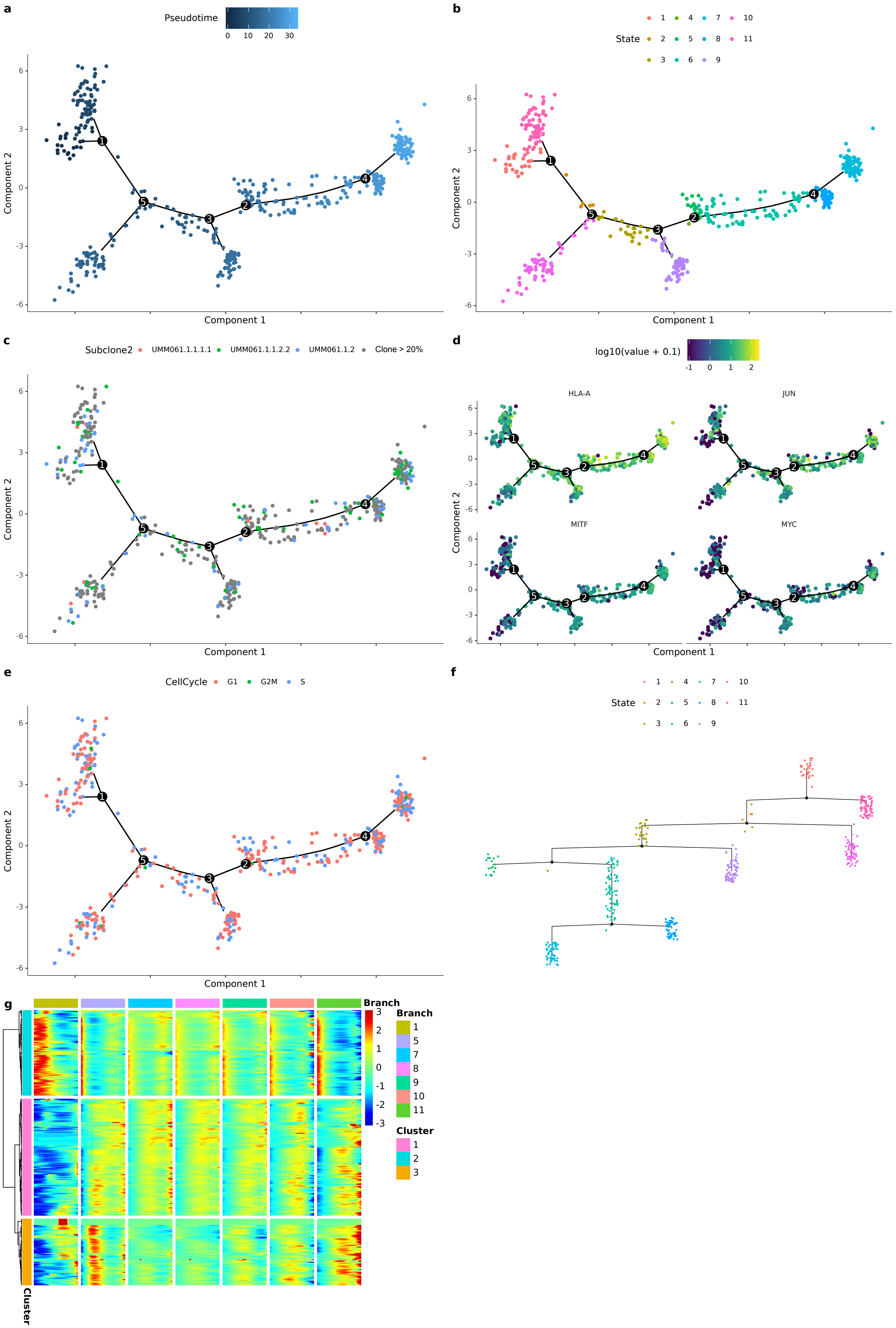
a
Pseudotime
0 10 20 30
Component 2
Component 1
b
State
1 2 3 4 5 6 7 8 9 10 11
c
Subclone2
UMM061.1.1.1.1
UMM061.1.1.2.2
UMM061.1.2
Clone > 20%
d
log10(value + 0.1)
HLA-A
JUN
MITF
MYC
e
CellCycle
G1
G2M
S
f
g
Branch
1 5 7 8 9 10 11
Cluster
1 2 3

UMM063

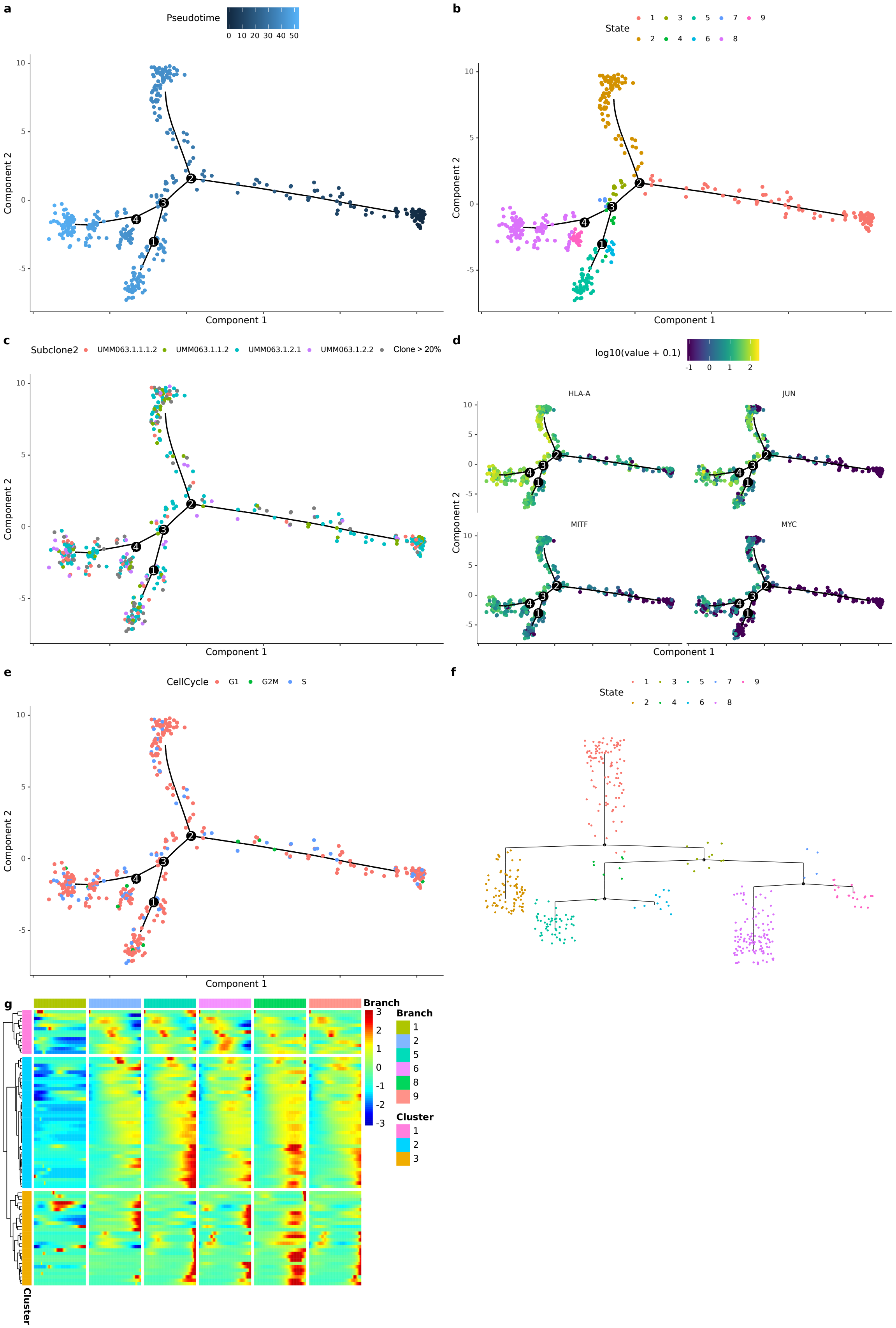

a
Pseudotime
0 10 20 30 40 50
Component 2
Component 1
b
State
1 2 3 4 5 6 7 8 9
c
Subclone2
UMM063.1.1.1.2
UMM063.1.1.2
UMM063.1.2.1
UMM063.1.2.2
Clone > 20%
d
log10(value + 0.1)
HLA-A
JUN
MITF
MYC
e
CellCycle
G1
G2M
S
f
g
Branch
1 2 5 6 8 9
Cluster
1 2 3

UMM064

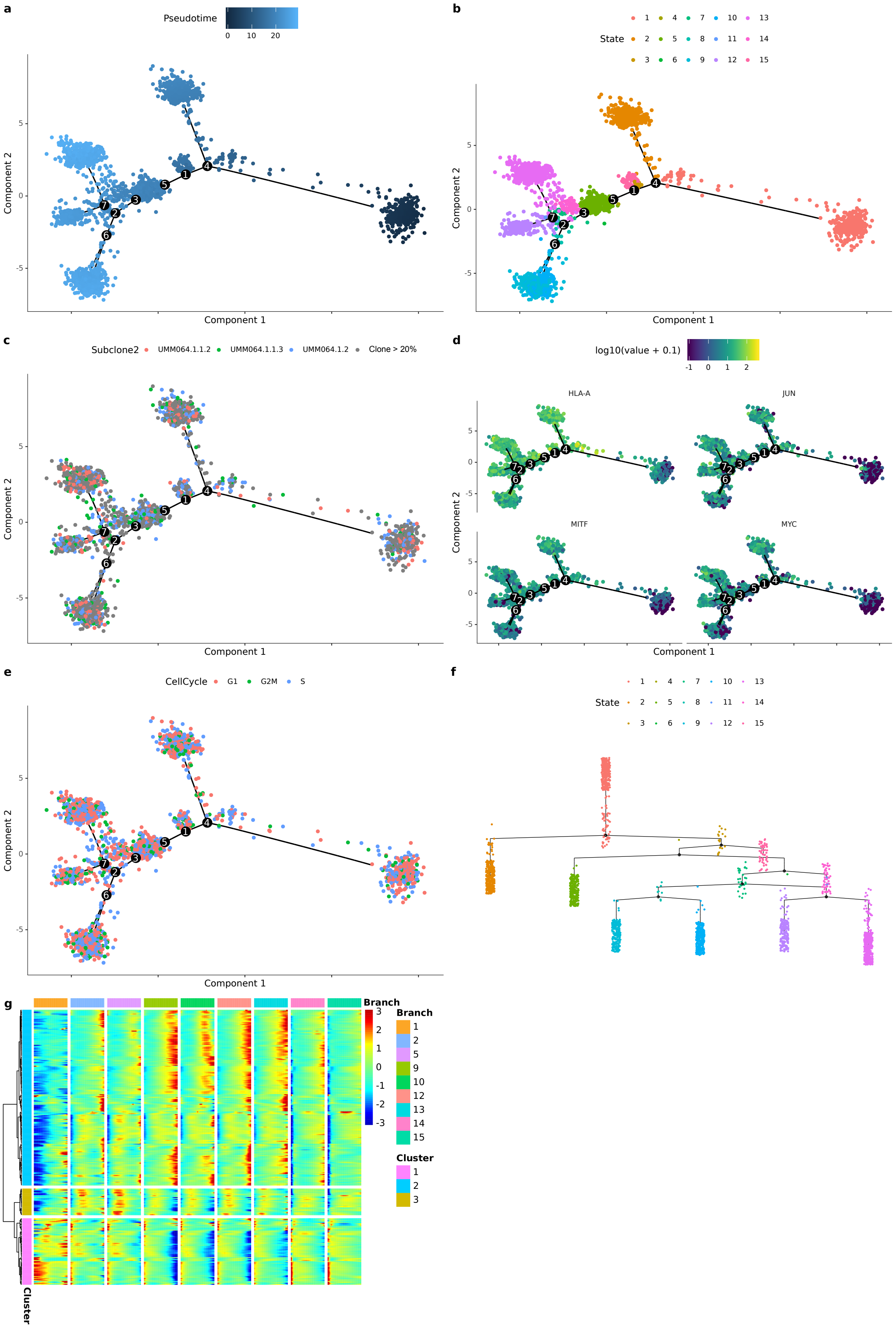

a
Pseudotime
0 10 20
Component 1
Component 2
b
State
1 2 3 4 5 6 7 8 9 10 11 12 13 14 15
c
Subclone2
UMM064.1.1.2
UMM064.1.1.3
UMM064.1.2
Clone > 20%
d
log10(value + 0.1)
HLA-A
JUN
MITF
MYC
e
CellCycle
G1
G2M
S
f
g
Branch
1 2 5 9 10 12 13 14 15
Cluster
1 2 3

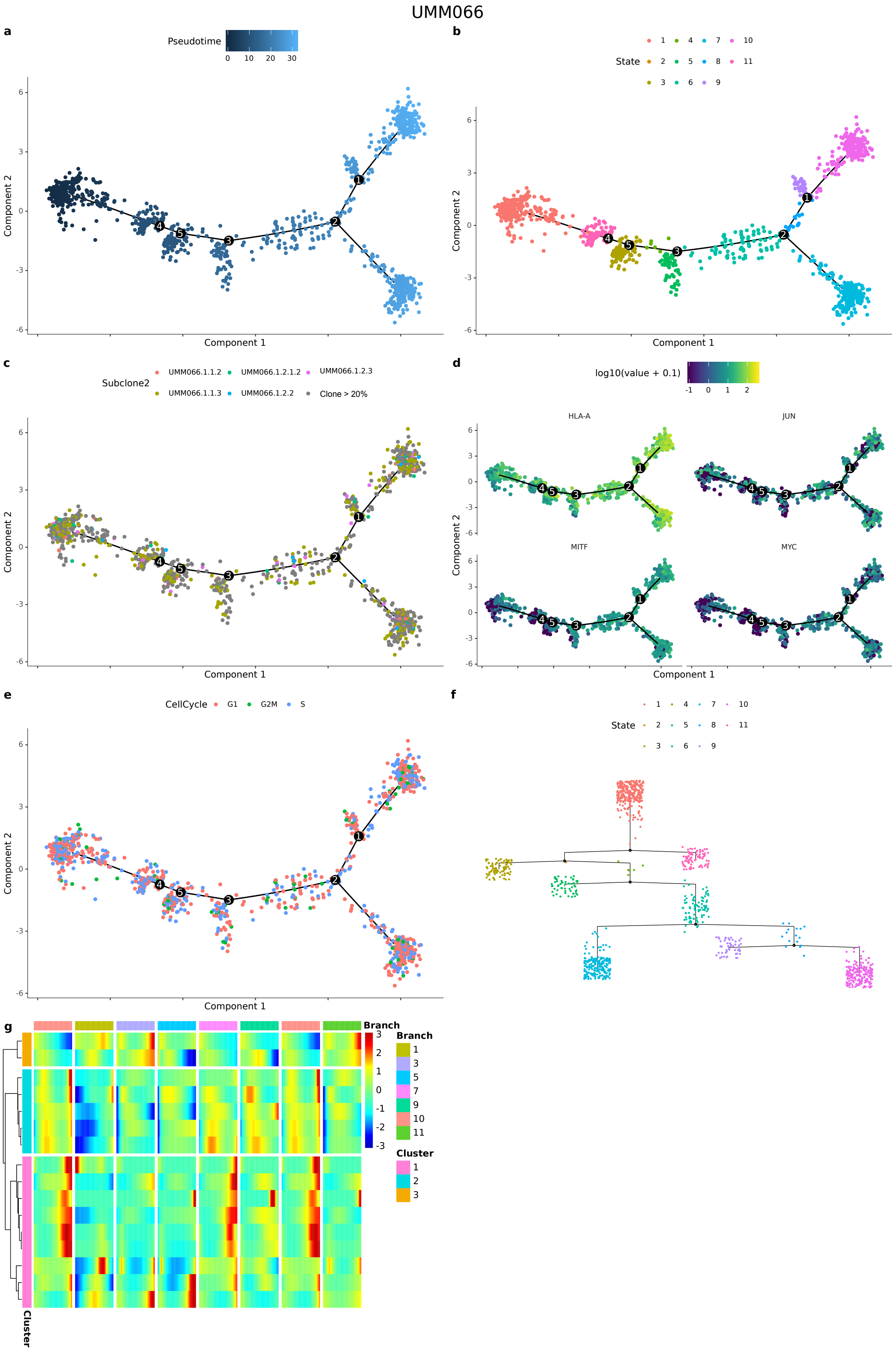

UMM066
a
Pseudotime
0 10 20 30
Component 2
Component 1
b
State
1 2 3 4 5 6 7 8 9 10 11
Component 2
Component 1
c
Subclone2
UMM066.1.1.2
UMM066.1.1.3
UMM066.1.2.1.2
UMM066.1.2.2
UMM066.1.2.3
Clone > 20%
Component 2
Component 1
d
log10(value + 0.1)
-1 0 1 2
HLA-A
JUN
MITF
MYC
Component 2
Component 1
e
CellCycle
G1
G2M
S
Component 2
Component 1
f
State
1 2 3 4 5 6 7 8 9 10 11
g
Branch
1 3 5 7 9 10 11
Cluster
1 2 3

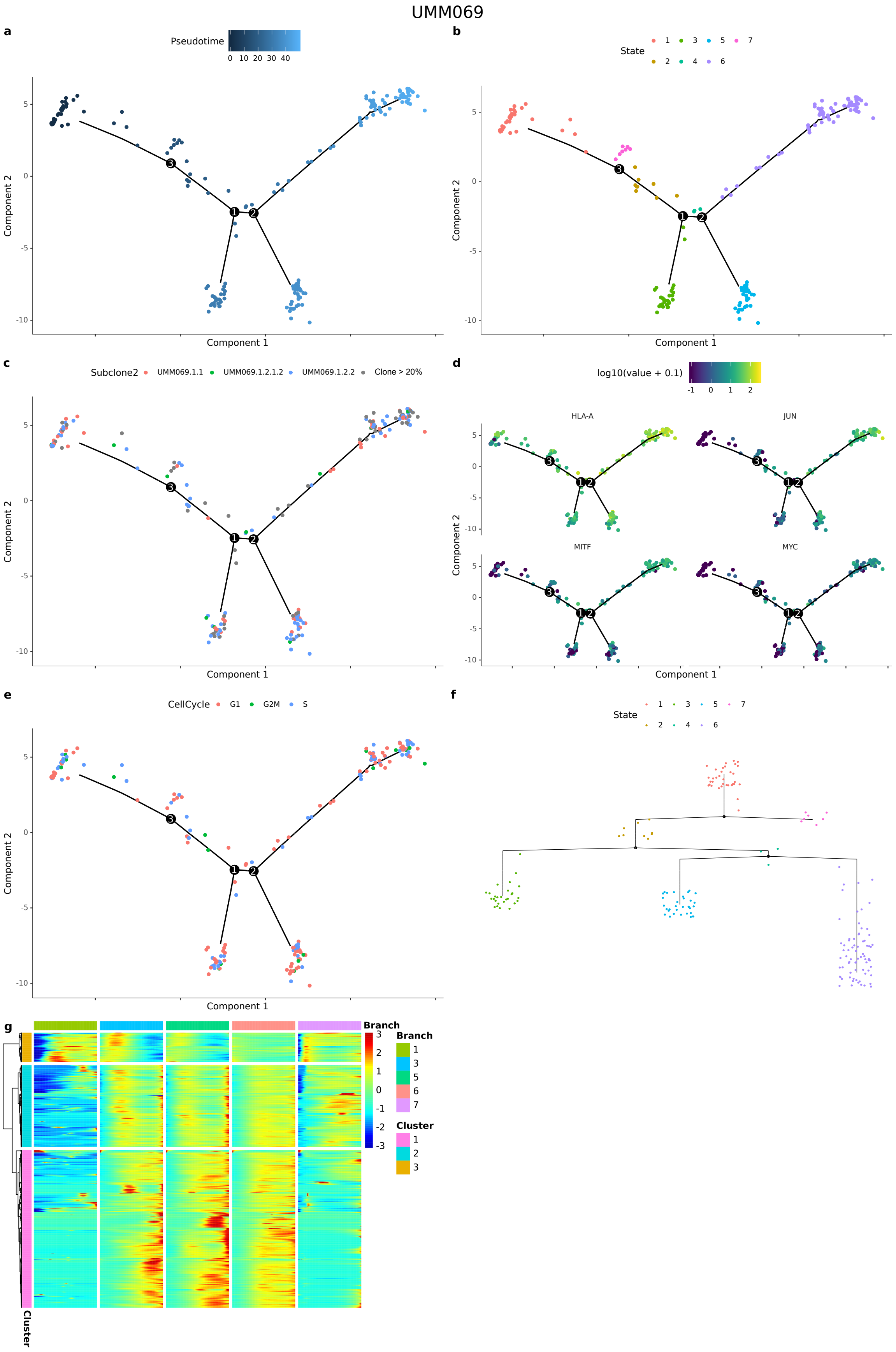
UMM069
a
Pseudotime
0 10 20 30 40
Component 2
Component 1
b
State
1 2 3 4 5 6 7
Component 2
Component 1
c
Subclone2
UMM069.1.1
UMM069.1.2.1.2
UMM069.1.2.2
Clone > 20%
Component 2
Component 1
d
log10(value + 0.1)
-1 0 1 2
HLA-A
JUN
MITF
MYC
Component 2
Component 1
e
CellCycle
G1
G2M
S
Component 2
Component 1
f
State
1 2 3 4 5 6 7
g
Branch
1 3 5 6 7
Cluster
1 2 3

UMM041L

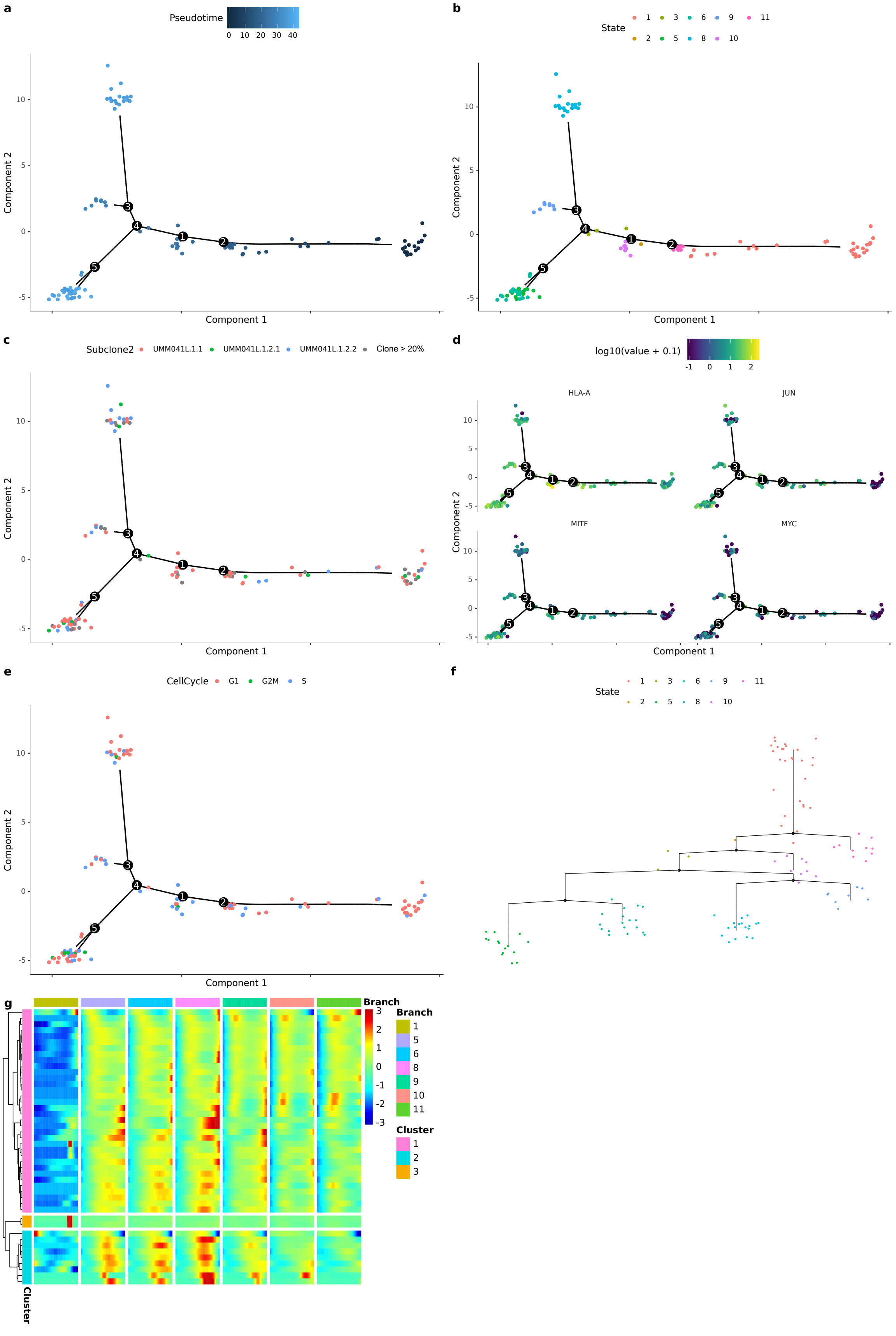

a
Pseudotime
Component 1
Component 2
b
State
c
Subclone2
UMM041L.1.1
UMM041L.1.2.1
UMM041L.1.2.2
Clone > 20%
d
log10(value + 0.1)
HLA-A
JUN
MITF
MYC
e
CellCycle
G1
G2M
S
f
g
Branch
Cluster

Supplementary Figure 6. Monocle 2 trajectory plots from individual primary and metastatic UM tumours. a, Trajectory plot coloured by pseudotime quantification. **b,** Trajectory plot annotated by calculated state. **c,** Trajectory plot annotated by calculated cell-cycle phase. **d,** Trajectory plot coloured by expression level of *JUN*, *MITF*, *HLA-A*, and *MYC* genes. *JUN*, *MITF*, *HLA-A*, and *MYC* are representative genes in their respective states (TNFA/NFKB, differentiation, HLA/immune, and MYC, respectively). *HLA-A* and *JUN* tend to differ most within each sample while changes in *MITF* and *MYC* are relatively minimal. **e,** Trajectory plot annotated by calculated cell-cycle phase. **f,** Complex cell trajectory plot with default root state showing the pseudotemporal ordering of the calculated cell states. **g,** Pseudotime heatmap of the genes determined from the BEAM analysis with a $q < 0.01$ distributed across the terminal branch states. Branch number is equivalent to state number in panels b and f.

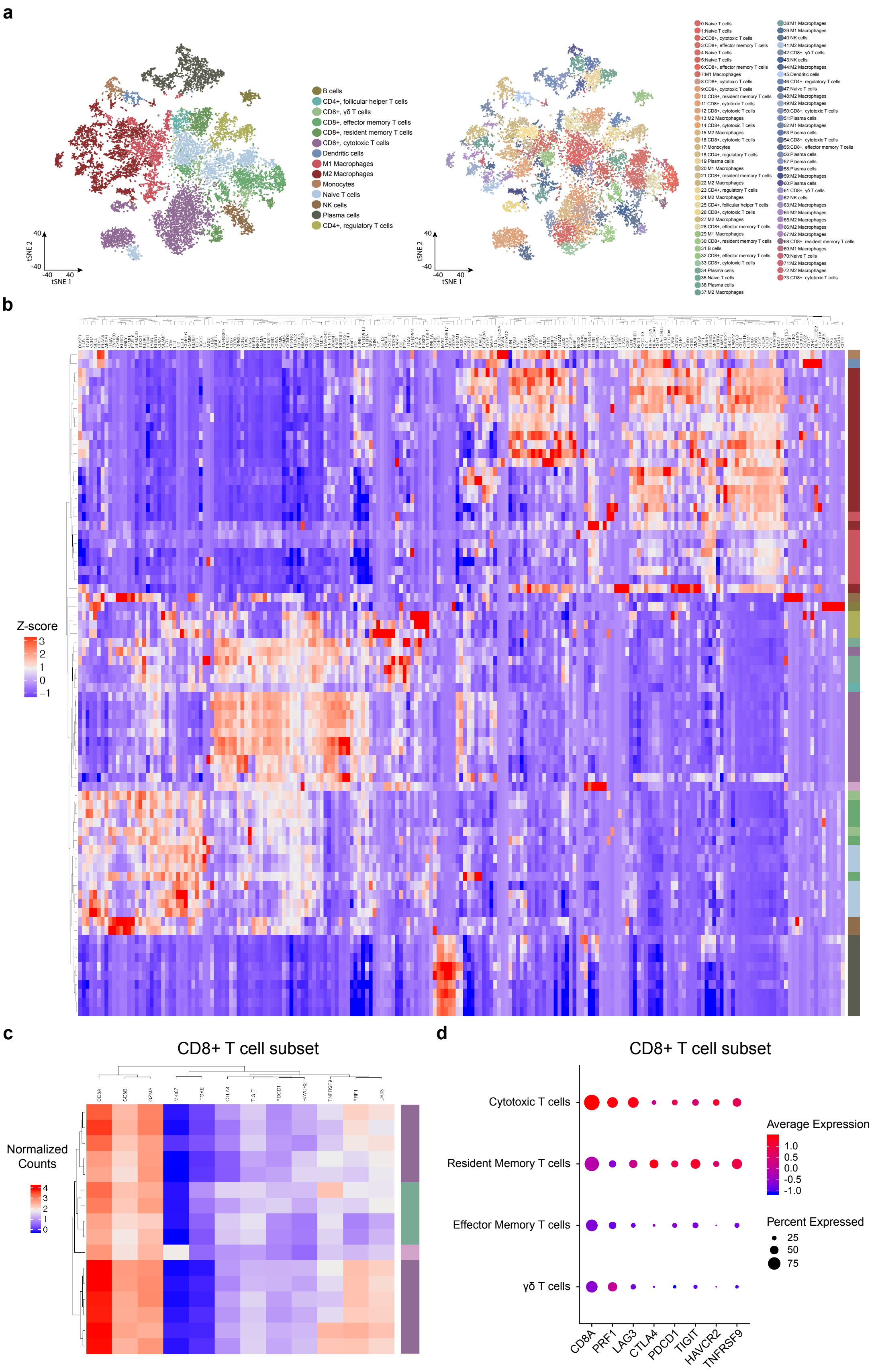
a
B cells
CD4+, follicular helper T cells
CD8+, γδ T cells
CD8+, effector memory T cells
CD8+, resident memory T cells
CD8+, cytotoxic T cells
Dendritic cells
M1 Macrophages
M2 Macrophages
Monocytes
Naive T cells
NK cells
Plasma cells
CD4+, regulatory T cells
tSNE 1
tSNE 2
b
Z-score
c
CD8+ T cell subset
Normalized Counts
d
CD8+ T cell subset
Cytotoxic T cells
Resident Memory T cells
Effector Memory T cells
γδ T cells
CD8A
PRF1
LAG3
CTLA4
PDCD1
TIGIT
HAVCR2
TNFRSF9
Average Expression
Percent Expressed

Supplementary Figure 7. Analysis of immune cell populations present in 8 primary and 3 metastatic uveal melanoma tumours. a, tSNE plot showing all of the annotated clusters identified using Louvain clustering. **b,** Heatmap with hierarchical clustering with an expanded selection of genes associated with various immune cells using Z-score and centered values. **c,** Heatmap of normalized counts of exhaustion markers (*PD1*, *CTLA4*, *TIM3*, *TIGIT* and *TNFRSF9*). **d,** Dot plot shows the higher percentage of CD8+, cytotoxic T cells expressing *LAG3* at higher levels of expression when compared to *PD1* and *CTLA4*. the size of the dot depicts the percentage of cells within each class, The colour intensity shows the AverageExpression() level of each class.

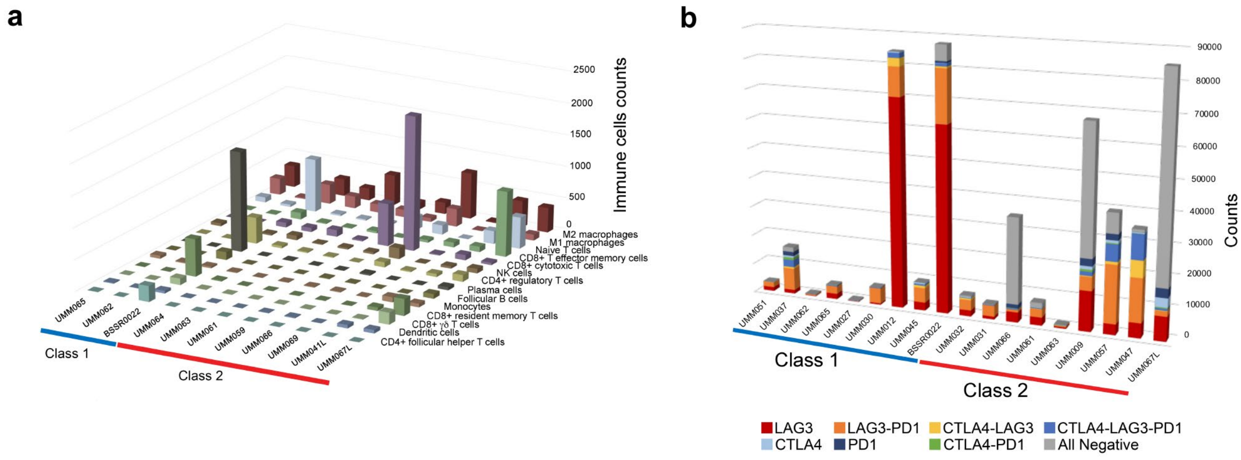

Supplementary Figure 8. Immune microenvironment of uveal melanomas. a, 3-dimensional bar chart of immune cell subtypes as a cell counts of immune cell populations for each tumour. **b,** Quantification of multi-colour IHC for CD8, LAG3, PD1, CTLA4, and DAPI as cell counts.

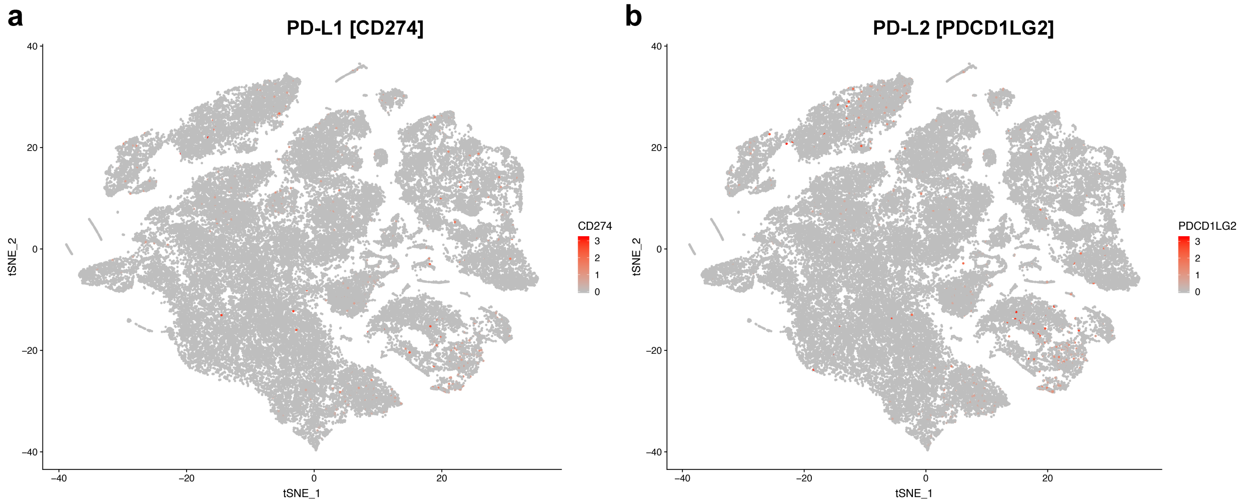

Supplementary Figure 9. PD-L1 and PD-L2 expression in 8 primary and 3 metastatic uveal melanoma tumours. a, Combined tSNE plot of *PD-L1* mRNA expression. **b,** Combined tSNE plot of *PD-L2* mRNA expression. Expression values are plotted as normalized counts.

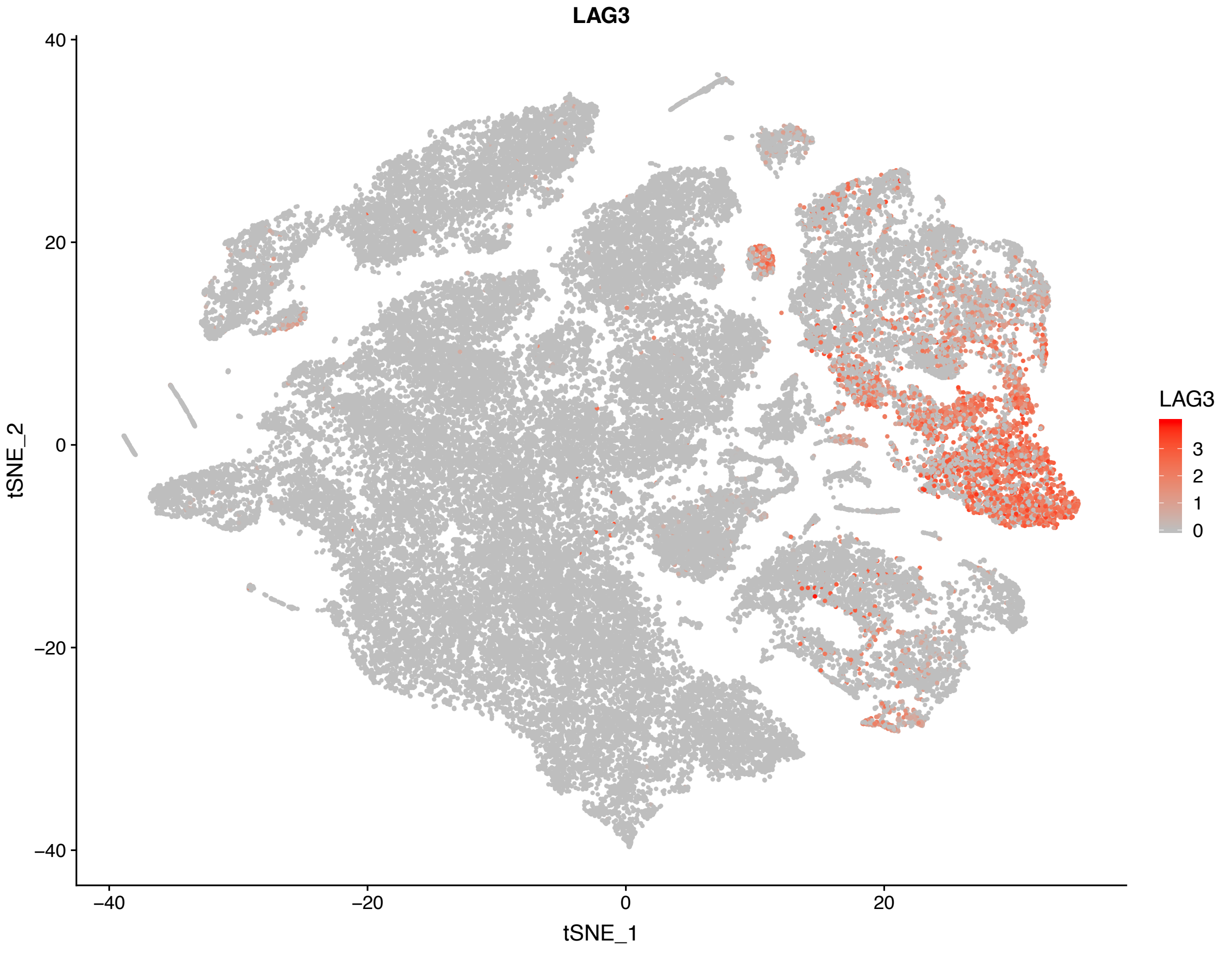

Supplementary Figure 10. LAG3 in 8 primary and 3 metastatic uveal melanoma tumours. Combined tSNE plot of *LAG3* mRNA expression. Expression values are plotted as normalized counts.

Supplementary Table 1. Clinical annotations, gene-expression profile, and *PRAME* status of 8 primary and 3 metastatic uveal melanomas.

Sample	Sample Type	GEP Class	PRAME	Age at Diagnosis	Sex	Tumour Diameter (mm)	Pathology Cell Type	Extrascleral Invasion	Ciliary Body Involvement	Metastasis	Months to Metastasis or Last Follow-Up
UMM059	Primary	2	Positive	86	F	15	Mixed	No	No	No	20
UMM061	Primary	2	Positive	71	F	19	Mixed	No	Yes	No	9
UMM062	Primary	1A	Negative	69	M	9	Mixed	No	No	No	1
UMM063	Primary	2	Negative	66	M	20	Mixed	Yes	Yes	Yes	13
UMM064	Primary	2	Positive	77	M	20	Mixed	No	Yes	No	2
UMM065	Primary	1A	Negative	44	F	10	Mixed	Yes	Yes	No	1
UMM066	Primary	2	Positive	53	M	18.4	Epithelioid	No	Yes	No	7
UMM069	Primary	2	Positive	80	F	19.8	Mixed	No	No	Yes	3
BSSR0022	Metastasis	1B	Positive	68	F	N/A	N/A	N/A	N/A	N/A	N/A
UMM067L	Metastasis	2	Positive	73	M	N/A	N/A	N/A	N/A	N/A	N/A
UMM041L	Metastasis	2	Positive	63	F	N/A	N/A	N/A	N/A	N/A	N/A

N/A, Not applicable; M, Male; F, Female.

Supplementary Table 2. Driver mutation status of 8 primary and 3 metastatic uveal melanomas.

Sample	Gaq Mutation	Gaq MAF	Gaq HGVS protein	BSE Mutation	BSE MAF	BSE HGVS protein	BSE Mutation Coverage
UMM059	GNA11	0.443	p.Gln209Leu	BAP1	0.669	p.Val616fs	964
UMM061	GNA11	0.493	p.Gln209Leu	BAP1	0.947	p.Ser123fs	262
UMM062	GNAQ	0.530	p.Gln209Pro	EIF1AX	0.994	p.Gly6splice	791
UMM063	GNAQ	0.434	p.Gln209Leu	BAP1	0.912	p.?	1711
UMM064	GNAQ	0.476	p.Gln209Pro	BAP1	0.883	p.Ser430fs	359
UMM065	GNA11	0.519	p.Gln209Leu	EIF1AX	0.531	p.Asn4Tyr	1668
UMM066	GNAQ	0.464	p.Gln209Pro	BAP1	0.746	p.Glu125splice	1062
UMM069	GNAQ	0.489	p.Gln209Leu	BAP1	0.958	p.Arg146splice	1815
BSSR0022	GNAQ	0.401	p.Gln209Pro	SF3B1	0.438	p.Arg625His	1249
UMM067L	GNA11	0.378	p.Gln209Leu	BAP1	0.527	p.Gly220splice	579
UMM041L	GNAQ	0.428	p.Gln209Pro	BAP1	0.438	p.Asp311fs	363

MAF, Mutant allele frequency; HGVS, Human Genome Variation Society; BSE, BAP1/Splicing/EIF1AX.

Supplementary Table 3. Cell types associated with the twelve gene expression profile genes.

Gene	Tumour class with increased expression by bulk analysis	Predominant cell type(s) expressing gene by single cell analysis
ID2	Class 1	T cells, macrophages, tumour cells
SATB1	Class 1	T cells
FXR1	Class 1	Tumour and immune cells
EIF1B	Class 1	Tumour and immune cells
LMCD1	Class 1	T cells, class 1B tumour cells
MTUS1	Class 1	Class 1A tumour cells
ROBO1	Class 1	Class 1A and 1B tumour cells
LTA4H	Class 1	Tumour and immune cells
HTR2B	Class 2	Class 2 tumour cells
RAB31	Class 2	Macrophages, class 2 tumour cells
ECM1	Class 2	Class 2 tumour cells
CDH1	Class 2	Class 2 tumour cells
