# Supplementary material for: Single-cell analysis reveals new evolutionary complexity in uveal melanoma
Source: Nat Commun. 2020 Jan 24;11:496. doi: 10.1038/s41467-019-14256-1 (PMC6981133; doi:10.1038/s41467-019-14256-1)
Supplement: Supplementary file 3 — Reporting Summary [file 41467_2019_14256_MOESM3_ESM.pdf]

nature research

Corresponding author(s): J. William Harbour

Last updated by author(s): Dec 18, 2019

Reporting Summary

Nature Research wishes to improve the reproducibility of the work that we publish. This form provides structure for consistency and transparency in reporting. For further information on Nature Research policies, see Authors & Referees and the Editorial Policy Checklist.

Statistics

For all statistical analyses, confirm that the following items are present in the figure legend, table legend, main text, or Methods section.

n/a	Confirmed	
☐	☒	The exact sample size (*n*) for each experimental group/condition, given as a discrete number and unit of measurement
☐	☒	A statement on whether measurements were taken from distinct samples or whether the same sample was measured repeatedly
☐	☒	The statistical test(s) used AND whether they are one- or two-sided<br>*Only common tests should be described solely by name; describe more complex techniques in the Methods section.*
☒	☐	A description of all covariates tested
☐	☒	A description of any assumptions or corrections, such as tests of normality and adjustment for multiple comparisons
☐	☒	A full description of the statistical parameters including central tendency (e.g. means) or other basic estimates (e.g. regression coefficient) AND variation (e.g. standard deviation) or associated estimates of uncertainty (e.g. confidence intervals)
☐	☒	For null hypothesis testing, the test statistic (e.g. *F*, *t*, *r*) with confidence intervals, effect sizes, degrees of freedom and *P* value noted<br>*Give P values as exact values whenever suitable.*
☒	☐	For Bayesian analysis, information on the choice of priors and Markov chain Monte Carlo settings
☒	☐	For hierarchical and complex designs, identification of the appropriate level for tests and full reporting of outcomes
☒	☐	Estimates of effect sizes (e.g. Cohen's *d*, Pearson's *r*), indicating how they were calculated

Our web collection on statistics for biologists contains articles on many of the points above.

Software and code

Policy information about availability of computer code

Data collection	No software was used for data collection.
Data analysis	General data analysis, statistical tests, and plotting: R (3.5.1,3.5.2) with the packages Seurat (2.3.4,3.0.0), ggplot2 (2.3.1,3.1.1), ComplexHeatmap (1.20.0), dplyr (0.7.8, 0.8.0.1), Rtsne (0.15), forcats (0.3.0, 0.40), bindrcpp (0.2.2), cowplot (0.9.3), Matrix (1.2-16), scales (1.0.0), jpeg (0.1-8), colorRamps (2.3), paletter (0.0.0.9000), cellranger (1.1.0), Monocle 2 (2.10.1), DDRTree (0.1.5), psycho (0.4.9), tidyverse (1.2.1), tibble (2.1.1), VGAM (1.1-1), irlba (2.3.3), stringr (1.4.0), Biobase (2.42.0), purrr (0.3.2), readr (1.3.1), ggpubr (0.2), DoubletFinder (2.0.2); louvain modularity optimizer by Ludo Waltman and Nees Jan van Eck (1.3); pySCENIC (0.9.9+2.gcaded79); GSEA/ MSigDB (v6.4); Leica Quantitative Algorithm (v1).<br>Single cell gene expression raw data processing Cellranger (10X Genomics, version 2.1.1).<br>Single cell VDJ raw data processing Cellranger (10X Genomics, version 2.2).<br>Single cell CNV raw data processing Cellranger dna (10X Genomics, version 1.0.0).

For manuscripts utilizing custom algorithms or software that are central to the research but not yet described in published literature, software must be made available to editors/reviewers. We strongly encourage code deposition in a community repository (e.g. GitHub). See the Nature Research guidelines for submitting code & software for further information.

Data

Policy information about availability of data

All manuscripts must include a data availability statement. This statement should provide the following information, where applicable:

- Accession codes, unique identifiers, or web links for publicly available datasets
- A list of figures that have associated raw data
- A description of any restrictions on data availability

All sequencing data generated have been deposited in dbGaP under accession code phs001861.v1.p1. Processed sequencing data have been deposited in GEO

under accession code GSE139829. The cisTarget Human motif database v9 used as part of the SCENIC analysis can be accessed at https://resources.aertslab.org/cistarget/motif2tf/motifs-v9-nr.hgnc-m0.001-o0.0.tbl. The source data underlying Figs 2b, 4d, and 4f are provided as a source data file.

Field-specific reporting

Please select the one below that is the best fit for your research. If you are not sure, read the appropriate sections before making your selection.

☒ Life sciences ☐ Behavioural & social sciences ☐ Ecological, evolutionary & environmental sciences

For a reference copy of the document with all sections, see nature.com/documents/nr-reporting-summary-flat.pdf

Life sciences study design

All studies must disclose on these points even when the disclosure is negative.

Sample size	This study was exploratory in nature and a pre-determined sample size was not specified. Cases were obtained as part of a prospective study. The final sample size was determined by the number of consecutive cases that became available in the clinical and surgical practices of study authors over a 9 month time period.
Data exclusions	No samples were excluded after single cell gene expression data was generated. No data was excluded from this analysis.
Replication	Characterization of cell populations present in the tumor micro-environment were confirmed by gene expression and histopathological determination. Selected markers were orthogonally validated by immunohistochemistry. Since these experiments have to be performed rapidly after eye removal the independent experiments were performed as the samples became available.
Randomization	Randomization is not applicable to our study design since no therapeutic intervention was evaluated.
Blinding	The author who conducted and quantified orthogonal validation with immunohistochemistry was blinded to sample identity. Blinding was not otherwise applicable to our study design since no therapeutic intervention was evaluated.

Reporting for specific materials, systems and methods

We require information from authors about some types of materials, experimental systems and methods used in many studies. Here, indicate whether each material, system or method listed is relevant to your study. If you are not sure if a list item applies to your research, read the appropriate section before selecting a response.

Materials & experimental systems

n/a	Involved in the study	
☐	☒	Antibodies
☒	☐	Eukaryotic cell lines
☒	☐	Palaeontology
☒	☐	Animals and other organisms
☐	☒	Human research participants
☒	☐	Clinical data

Methods

n/a	Involved in the study	
☒	☐	ChIP-seq
☒	☐	Flow cytometry
☒	☐	MRI-based neuroimaging

Antibodies

Antibodies used	UltraPlex antibodies used in this study from Cell IDx were obtained directly from Epitomics, a subsidiary of Abcam. They provide formulations better suited for Cell IDx's manufacturing purposes. Since antibodies were not obtained directly from the Abcam website they do not have an Abcam catalog numbers. Abcam numbers corresponding to antibodies generated using the same DNA sequences are provided where available. The clone and lot number of the antibodies are provided as follows: CTLA-4-CH014 (Clone CAL49, Lot 121617), CD8-CH015 (Clone EP334, Lot 19020512, Abcam: EPR10640(2)), PD-1-CH016 (Clone EP239, Lot 19020511, Abcam: EPR4877(2)), and LAG3-CH021 (Clone EP294, Lot EP092402, Abcam: EPR4392(2)).
Validation	The antibodies in the UltraPlex panel were obtained from Abcam/Epitomics and then, prior to incorporating into UltraPlex panels, they were internally qualified at Cell IDx by staining human tonsil with these antibodies by IHC. These images were then reviewed by a pathologist to ensure that they showed the appropriate staining pattern for their biomarker specificity.<br>Cell IDx has developed a proprietary, modified hapten-based technology to address the need for unrestricted, simple, rapid, and simultaneous detection of multiple cellular markers in tissue sections. Chromogenic detection of single markers can no longer keep pace with the requirement for detailed information on cell subpopulations, levels of marker expression per cell, and individual cellular localization in situ relative to other cells. To address these issues, we have developed this new technology with inspiration from flow-cytometry's ability to phenotype and quantitate small subpopulations of cells identified by simultaneous expression of several markers, and translated this multiplex staining to tissue sections. Directly-labeled antibodies, such as are used in flow-cytometry, generally give weak staining in tissue sections compared to use of fluorescent-labeled secondary antibodies. However, use of fluorescent secondaries is limited by the availability of primary antibodies of different species or isotypes to avoid cross-reactivity. Cell IDx has developed an expanding range of next-generation, modified haptens which we use

to label primary antibodies. Primary antibodies are combined in cocktails of four antibodies and then detected with a panel of anti-hapten secondary antibodies each labeled with a different fluor. The result is a simple two-hour, two-step staining procedure yielding the type of data previously impossible.

Human research participants

Policy information about studies involving human research participants

Population characteristics	Total patients with data n = 11<br>Total samples with data n = 11<br>Female n = 6<br>Male n = 5<br>Primary tumours n = 8<br>Liver metastases n = 3<br><br>Primary tumours<br>Class 1A: n =2<br>Class 2: n = 6<br>Mean age: 68.25 (min 44 - max 86)<br>Mean tumour LBD: 16.4 mm (min 9mm- max 20mm)<br><br>Metastatic tumours<br>Class 1B: n = 1<br>Class 2: n = 2<br>Mean age: 68 (min 63 - max 73)
Recruitment	Patients enrolled in this study were recruited by the treating physician and signed informed consent. There were no self-referral patients as part of this study. Patients were recruited based on current diagnosis of uveal melanoma and treatment with enucleation or liver resection for removal of primary or metastatic uveal melanoma. Care was taken to minimize bias by taking consecutive cases and using single-cell RNAseq, an agnostic approach to data analysis.
Ethics oversight	University of Miami Institutional Review Board

Note that full information on the approval of the study protocol must also be provided in the manuscript.
