# Supplementary material for: Single-cell analysis reveals new evolutionary complexity in uveal melanoma
Source: Nat Commun. 2020 Jan 24;11:496. doi: 10.1038/s41467-019-14256-1 (PMC6981133; doi:10.1038/s41467-019-14256-1)
Supplement: Supplementary file 4 — Description of Additional Supplementary Files [file 41467_2019_14256_MOESM4_ESM.pdf]

Description of Additional Supplementary Files

File Name: Supplementary Data 1
Description: Significant genes identified using the two-sided non-parametric Wilcoxon rank sum test for each annotated cell cluster from the analyzed 59,915 cells.

File Name: Supplementary Data 2
Description: Results of MSigDB analysis of BEAM genes for each individual tumour hierarchical cluster. Statistical analysis was performed using one-sided hypergeometric test with correction for multiple hypothesis testing according to Benjamini and Hochberg.

File Name: Supplementary Data 3
Description: Significant genes identified using the two-sided non-parametric Wilcoxon rank sum test for each annotated immune cell cluster.
